# Management and outcomes of obstructive sleep apnea in children with Robin sequence, a cross-sectional study

**DOI:** 10.1007/s00784-016-1985-y

**Published:** 2016-11-26

**Authors:** Manouk J.S. van Lieshout, Koen F.M. Joosten, Maarten J. Koudstaal, Marc P. van der Schroeff, Karolijn Dulfer, Irene M.J. Mathijssen, Eppo B. Wolvius

**Affiliations:** 1 Department of Oral and Maxillofacial Surgery, Erasmus Medical Center, Sophia Children’s Hospital, Room D-210,, 3000 CA Rotterdam,, The Netherlands; 2 Dutch Craniofacial Center, Erasmus MC, Rotterdam, The Netherlands; 3 Department of Pediatrics, Intensive Care Unit, Erasmus Medical Center, Sophia Children’s Hospital, Rotterdam, The Netherlands; 4 Department of Otorhinolaryngology and Head and Neck Surgery, Erasmus Medical Center, Sophia Children’s Hospital, Rotterdam, The Netherlands; 5 Department of Child and Adolescent Psychiatry/Psychology, Erasmus MC, Sophia Children’s Hospital, Rotterdam, The Netherlands; 6 Department of Plastic, Reconstructive and Hand Surgery, Erasmus MC, Sophia Children’s Hospital, Rotterdam, The Netherlands

**Keywords:** Robin sequence, Airway obstruction, Obstructive sleep apnea, Treatment

## Abstract

**Objective:**

The objective of this cross-sectional study is to assess the prevalence, course, and management of obstructive sleep apnea (OSA) in children with Robin sequence (RS) aged 1–18 years.

**Materials and methods:**

A cross-sectional study was conducted in 63 children aged 1 to18 years with RS. Patient data were collected on baseline characteristics and management. OSA was evaluated by polysomnography.

**Results:**

Sixty-three children with RS were included (median age 8.0 years) and divided into two groups based on the initial treatment: prone positioning or respiratory support. Respiratory support was more often indicated in children with a non-isolated RS (*p* < 0.05). At cross section, in the prone positioning group (*n* = 32), one child was diagnosed with OSA. In the respiratory support group (*n* = 31), 13 children (42 %) had respiratory problems of whom 10 needed respiratory support.

**Conclusions:**

Between the age of 1 and 18 years, almost one out of four children with RS still has respiratory problems. Children with RS, who can be treated with prone positioning only as an infant, are not likely to develop obstructive airway problems at a later age. In contrast, children who need respiratory support early after birth are at risk of continuing or re-developing OSA after the age of 1 year.

**Clinical relevance:**

This study shows that those who need respiratory support at an early age need careful monitoring until adulthood.

## Introduction

Robin sequence (RS) is a congenital facial condition occurring in 1 in 5600 to 1 in 30,000 newborns [1–5]. The condition is classically characterized by an underdeveloped mandible (mandibular hypoplasia), backward displacement of the tongue (glossoptosis), and airway obstruction. In 80–90 % of the RS cases, a cleft palate is present [4, 6, 7].

Children with RS are at risk of developing obstructive sleep apnea (OSA) [8]. OSA is characterized by prolonged partial upper airway obstruction and/or intermittent complete airway obstruction, disrupting the child’s sleeping pattern [9]. Leaving OSA untreated may result in serious morbid consequences on the cardiovascular system, the metabolic system, and neurocognitive and behavioral functioning [9]. To establish the presence of OSA, polysomnography (PSG) is currently considered the gold standard [10].

The prevalence of respiratory problems in infants with RS is considerably high with reported OSA prevalence rates between 46 and 100 % depending on the criteria used [11, 12, 8, 13, 14]. However, to our knowledge, no follow-up studies on OSA have been conducted in children with RS beyond the infant period.

The aim of this cross-sectional study was to examine the prevalence, course, and management of OSA in children over the age of 1 year with RS.

## Material and methods

A cross-sectional study was carried out among children with RS aged above 1 year combined with retrospective data collection from the patients’ chart. Inclusion was based on historical data of the Dutch Craniofacial Center, Erasmus Medical Centre-Sophia’s Children Hospital. Children were considered suitable for inclusion if they had been diagnosed with RS, in this study defined as the presence of mandibular hypoplasia and airway obstruction, and were aged between 1 and 18 years [15, 16]. The ethical committee of the Erasmus Medical Centre (MEC-2012-048) approved the study. For all participating children, parents and/or children (if above 12 years of age) provided written informed consent. As primary treatment providers, no approval of the ethical committee was necessary for the data collection of baseline characteristics of the non-participants. Inclusion and study visits took place between November 2012 and July 2015.

During the study visit, participants underwent PSG at home or in the hospital. Furthermore, a retrospective chart review was performed on the initial treatment of airway obstruction from birth on until cross section. Based on these retrospective data, children were divided into two groups: those who initially had been treated with prone positioning and those who initially had been treated with respiratory support. Interventions of airway obstruction, which were performed in other centers, were also taken into account in this review. PSG results and treatment history were analyzed for these two groups.

### Polysomnography

In this study, ambulant sleep studies (level III, with data recorded by the Embletta portable diagnostic system) and clinical sleep studies in the hospital (level I, i.e., attended PSG including medical and technical support) were done. During the sleep studies, a variety of cardiorespiratory variables were assessed, including nasal airflow, chest and abdominal wall motion, and arterial oxygen saturation. Data were analyzed using Somnologica for Embletta software 3.3 ENU (Medcare Flags, Reykjavik, Iceland) for ambulant studies and Shell+ BrainRT Software Suite Version 2.0 (O.S.G., Rumst, Belgium) for clinical studies.

For analysis, we aimed for a total sleep time (TST) of at least 360 min, free of artifact. Summary statistics and events were scored according to the updated rules for scoring respiratory events by the American Academy of Sleep Medicine (AASM) [17]. An obstructive event was defined as a reduction in nasal airflow of ≥90 % (apnea) or 30–90 % (hypopnea) for the duration of at least two breaths, in the presence of thoracic and abdominal breathing movement. A hypopnea was only included if it was associated with a subsequent SpO_2_ desaturation of at least 3 % from baseline or with an arousal. Central apnea/hypopnea meets the same criteria as its obstructive counterpart, only without the presence of thoracic and abdominal breathing movement. A mixed apnea is a combination of a central apnea and an obstructive apnea. The obstructive apnea–hypopnea index (oAHI) was calculated by adding the number of obstructive apneas, mixed apneas, and obstructive hypopneas with SpO_2_ desaturation, divided by the TST; OSA was defined as an oAHI ≥1 per hour. An oAHI ≥1 and <5 was defined as mild OSA, between ≥5 and <25 as moderate OSA, and ≥25 as severe OSA [18].

### Statistical analysis

To assess whether the participant group was not significantly different from the non-participant group, baseline characteristics were compared using Pearson’s chi-squared tests and independent Student *t* tests. In order to determine the mean age at time of cross section in the group of non-participants, we used the date halfway our study inclusion period as date of cross section. A *p* value of <0.05 was considered to be statistically significant. Analyses were performed with SPSS 20.0 for Windows (SPSS, Inc. Chicago, IL).

## Results

### Baseline characteristics

**Fig. 1:**
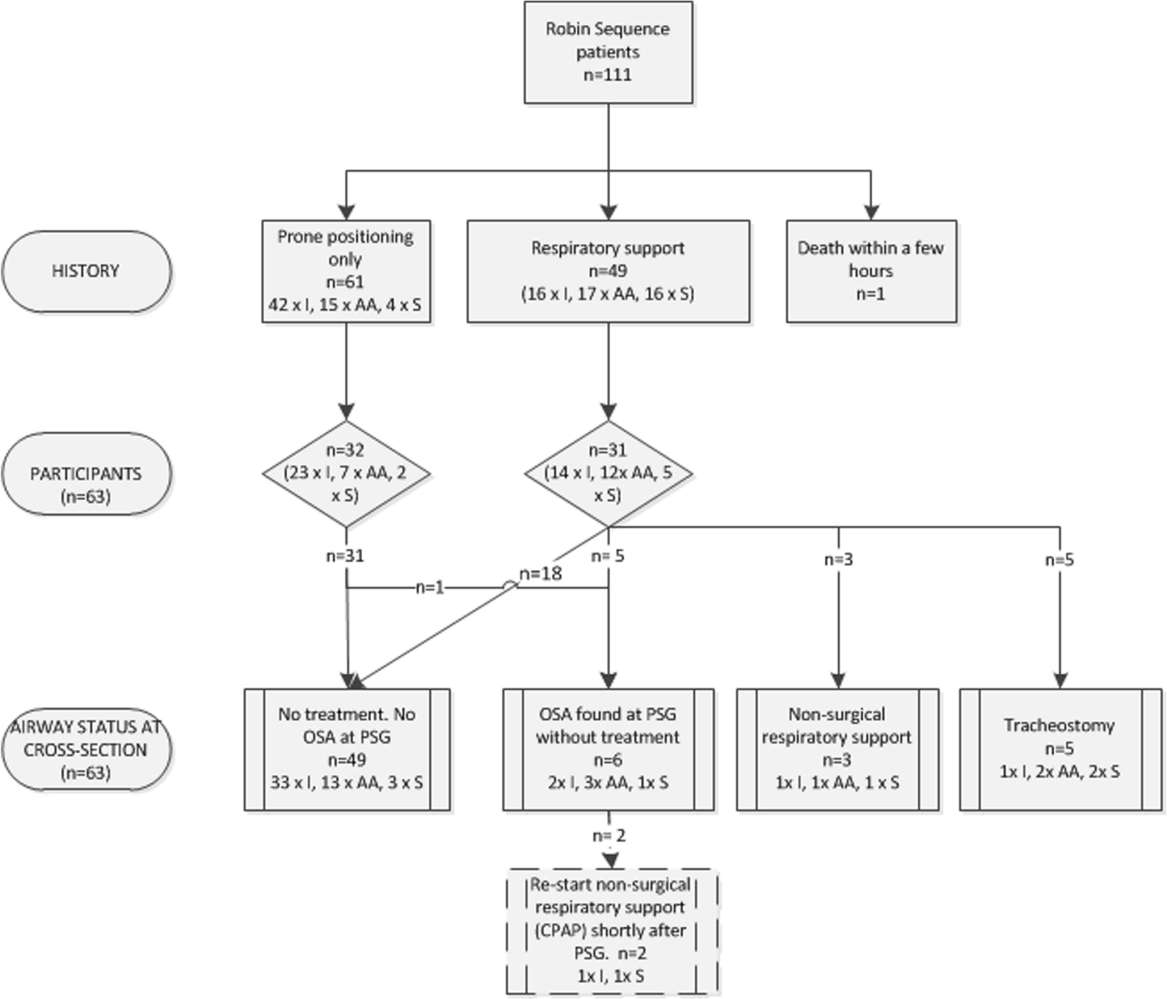
Treatment overview of children with RS. *I* isolated, *AA* associated anomalies, *S* proven syndrome

In total, 111 children with RS were eligible for inclusion of whom 63 (57 %) RS patients and their parents gave informed consent (Fig. 1). For 48 children, consent was not obtained due to various reasons. In order to assess whether the study sample was representative for the RS population in our hospital, in Table 1, baseline characteristics of the study participants are compared with the non-participants. No significant differences were found for mean age at cross section, sex, presence of a syndrome or additional anomalies, presence of a cleft palate, and initial treatment of airway obstruction.

**Table 1:** Baseline characteristics of the study participants (*n* = 63) vs. non-participants (*n* = 48)

		Participants (63)	Non-participants (*n* = 48)	*p* value
Median age in years at cross section (IQR)		8.0 (4.0–12.0)	9.0 (6.0–13.0)	*p* = 0.57
Sex	Female Male	31 (49.2 %) 32 (50.8 %)	21 (43.8 %) 27 (56.3 %)	*p* = 0.57
Presence of a syndrome or additional anomalies	Yes, additional anomalies Yes, syndrome No	19 (30.2 %) 7 (11.1 %) 37 (58,7 %)	14 (29.2 %) 13 (27.1 %) 21 (43.8 %)	*p* = 0.08
Presence of a cleft palate	Yes No	58 (92.1 %) 5 (7.9 %)	40 (83.3) 8 (16.7 %)	*p* = 0.16
Treatment of airway obstruction	Prone positioning only Non-surgical treatment Surgical treatment	32 (50.8 %) 19 (30.2 %) 12 (19.0 %)	29 (60.4 %) 8 (16.7 %) 11 (22.9 %)	*p* = 0.19

*IQR* interquartile range

Five children were deceased but were nonetheless included in the calculations of the non-participation group. In two of these cases, the cause of death was respiratory-related: one child with various comorbidities died at the age of 1 year due to severe respiratory insufficiency following aspiration and another child died within hours after birth due to severe obstruction of the upper airway and no option for a tracheostomy.

Thirty-one females and 32 males participated in the study. Of these 63 children, 37 children had an isolated RS, while 26 children had additional anomalies (*n* = 19) or a syndrome (*n* = 7); 2 children were diagnosed with Stickler syndrome, 1 child with Nager syndrome, 1 child with Shprintzen–Goldberg syndrome, 1 child with acampomelic dysplasia, 1 child with a FOXC2 mutation, and 1 child with a MFDM mutation. A variety of conditions were reported in the group with associated anomalies such as psychomotor retardation, hip dysplasia, or facial anomalies, but not with a proven syndrome. Five children had RS without a cleft palate.

The mean pregnancy duration was 38.5 weeks. Eight children were born pre-term (<37 weeks). The mean birth weight was 3137 g. Three families reported occurrence of mandibular hypoplasia in the family.

Feeding difficulties were reported in 60 out of 63 (95.2 %) children. Twenty-six children out of 63 (41.3 %) needed a temporary feeding tube and seven children (11.1 %) a percutaneous endoscopic gastrostomy tube.

### Prior to cross section: initial management of obstructive sleep apnea

**Fig. 2:**
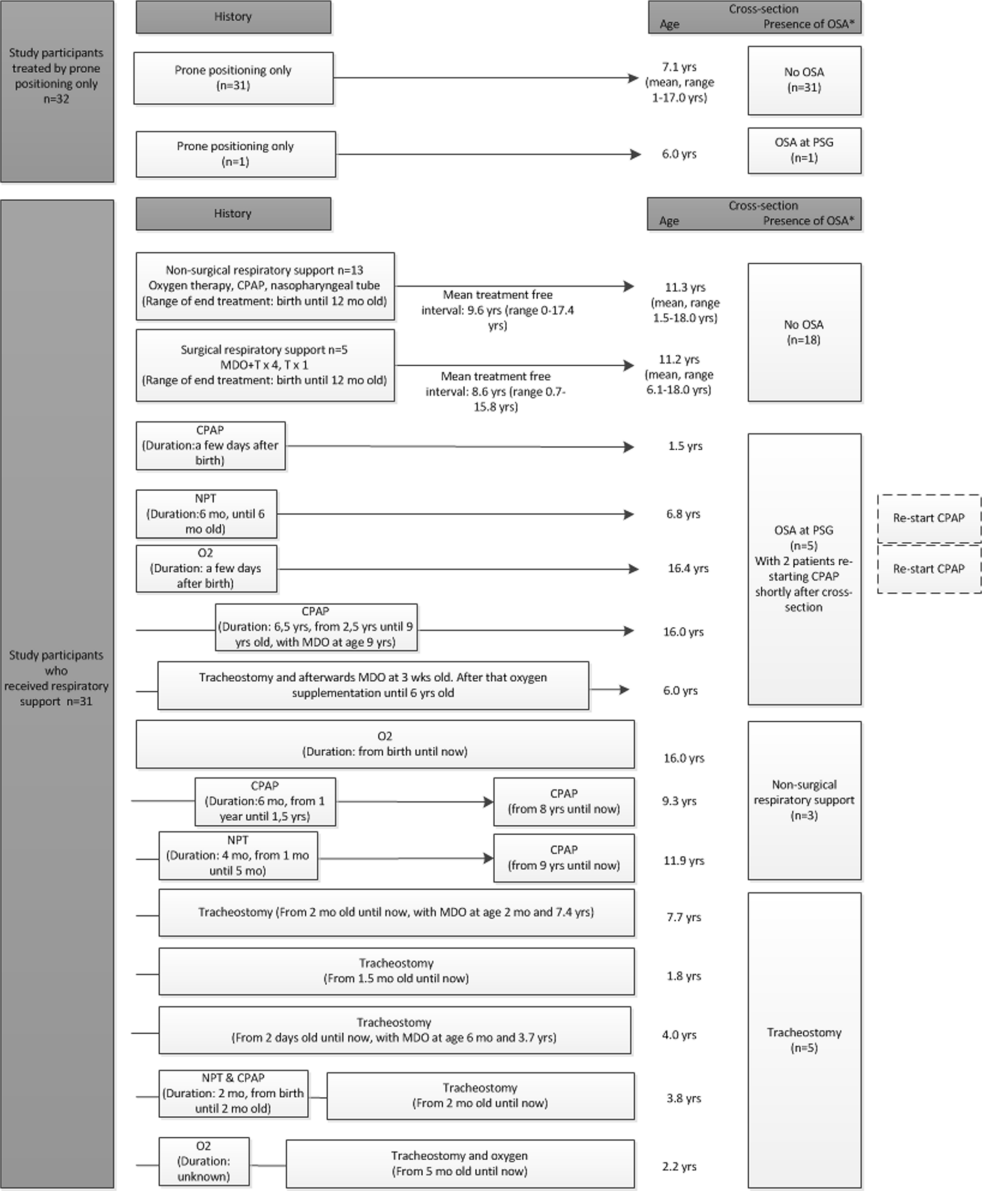
Treatment overview of study participants (*n* = 63) including the (mean) follow-up duration and age at cross section. *MDO* mandibular distraction osteogenesis, *T* tracheostomy, *yrs* age in years, *mo* age in months. Presence of OSA was based on PSG results (*n* = 44) (*asterisk*). If no PSG was available, presence of OSA was based on the need for treatment

Figure 2 shows an overview of initial management (including the findings at cross section). Initially, 32 children (48.5 %) were treated with prone positioning and 31 children were in need of respiratory support, which consisted of non-surgical respiratory support such as a nasopharyngeal tube, continuous positive airway pressure (CPAP), and oxygen therapy, or surgical measures such as tracheostomy and mandibular distraction osteogenesis with or without tracheostomy at the time of distraction. Children with additional anomalies or a syndrome (*n* = 26) were in need of respiratory support significantly more often compared to those with an isolated RS (65.4 vs. 37.8 %, *p* < 0.05).

### At cross section

The median age in years at cross section was 8.0 years (IQR 4.0–12.0). Thirty-one out of 63 (49.2 %) children were female. Thirty-seven children (58.7 %) had an isolated RS. A cleft palate was present in 58 (92.1 %) children.

### At cross section: assessment of obstructive sleep apnea by polysomnography

From the 63 children in this study, in 19 no PSG result was available, in 3 the parents refused, in 10 PSG failed due to logistic reasons, and in 6 PSG was not indicative of OSA because of a tracheostomy (*n* = 5) or CPAP (*n* = 1). These last six cases were classified as having OSA without formal assessment. At the end, 44 PSG studies (*n* = 37 ambulatory and *n* = 7 clinical) were available for analysis: 23 PSG studies in the group initially managed with prone positioning (*n* = 32) and 21 PSG studies in the group initially managed with respiratory support (*n* = 31).

OSA was detected in six children who did not receive OSA treatment at the time of cross section. Besides these six children, OSA was confirmed in two other children who already received respiratory support for OSA but a PSG was done without this support as part of routine clinical evaluation. Table 2 further elaborates on these cases.

**Table 2:** Overview of cases in whom OSA was found during PSG (*n* = 44)

Case number, isolated or non-isolated, age at cross section	History	PSG at cross section	
		oAHI	ODI
Case 1, isolated, 6.0 years	Treated by prone positioning in the neonatal period and no complaints afterwards. After PSG, the child was treated with nasal corticosteroids because of mild–moderate OSA.	3.7	2.8
Case 2, associated anomalies, 1.5 years	PSG for follow-up purposes and a wait-and-see policy was set. This child was already known to have OSA and hypoventilation.	6.7	9.5
Case 3, syndromal, 6.8 years	A nasopharyngeal tube shortly after birth for 5 months. After PSG, CPAP was re-started because of moderate OSA.	17.0	20.9
Case 4, isolated, 16.4 years	A few days of oxygen therapy at birth and afterwards treated with CPAP for 3 months. At the age of 16, severe complaints of OSA and re-start of CPAP shortly after PSG	50.0	26.7
Case 5, associated anomalies, 16.0 years	CPAP for 6.5 years until the age of 9 years, when mandibular distraction osteogenesis was performed. Based upon the PSG results, a wait-and-see policy was set.	3.2	0.7
Case 6, associated anomalies, 6.0 years	Mandibular distraction osteogenesis and decannulation at the age of 3 months and oxygen supplementation until the age of 6 years. Based upon the PSG results, a wait-and-see policy was set.	7.3	8.9
Case 7, isolated, 9.3 years	Six months of CPAP treatment at the age of 1 year and re-start of CPAP at the age of 8 years. At home PSG without CPAP	3.0	0.2
Case 8, syndromal, 16.0 years	Oxygen therapy since birth, at home PSG without oxygen	7.0	3.7

### At cross section: airway management

Out of the total group of 63 children, 14 (22 %) received either OSA treatment and/or showed OSA during PSG at cross section.

In the cohort of the children (*n* = 32, mean age at cross section 7.1 ± 4.5 years) who were initially treated with prone positioning, in one child mild OSA was found with PSG, but further treatment was not necessary. Thirty-one out of 32 (96.9 %) children were free of OSA treatment at the time of cross section.

In the cohort of children (*n* = 31, mean age at cross section 9.4 ± 5.8 years) who were initially treated by respiratory support, 13 children (42 %) had respiratory problems; 3 children were still in need of non-surgical respiratory support (CPAP or oxygen therapy) (age range at cross section 9.3–16.0 years), and 5 children were dependent on a tracheostomy (age range at cross section 1.8–7.7 years). In five children, OSA was found (age range 1.5–16.4 years), and in two of these children a re-start of respiratory support (CPAP) was indicated shortly after PSG (Table 2). Eighteen out of 31 (58.1 %) children were free of OSA or respiratory support at the time of cross section.

## Discussion

To our knowledge, this is the first long-term follow-up study investigating obstructive airway problems in children with RS between 1 and 18 years of age. At cross section, 22 % of the children with RS still had respiratory problems. Those who had a history of only prone positioning were not likely to develop a significant airway obstruction at a later age. However, children who needed respiratory support early after birth were 13 times more likely to be diagnosed with OSA at a later age or to remain dependent on or re-develop a need for respiratory support.

In this study, children were divided into two groups on the basis of their initial airway management. About half of the children initially needed respiratory support for which different respiratory support modalities were used. In eight children, mandibular distraction osteogenesis had been performed. At cross section, in six of these children mandibular distraction osteogenesis was successful, although in two cases mild OSA was diagnosed with PSG but no further treatment was necessary. Two children still needed a tracheostomy at the time of the study; however, follow-up after the mandibular distraction osteogenesis was short. Also, two children who had received non-surgical respiratory support shortly after birth were still dependent on a tracheostomy.

Interestingly, four children became again in need of non-surgical respiratory support at an older age, in two of them as a result of this study. In a recent retrospective study of Lee et al., it was shown that their RS infant population, who were followed to 1 year of age, did not show significant decreases in AHI, oAHI, and central apnea index [19]. Unfortunately, other studies with follow-up on OSA in RS patients are so far lacking. On the basis of this study, one might conclude that the group of children who initially needed respiratory support might benefit from more careful monitoring until adulthood.

Remarkably, of those who were still in need of respiratory support, six children had been diagnosed with associated anomalies or a syndrome and only two with an isolated RS. In daily practice (data not shown), many children with an associated anomaly or a syndrome have an even smaller mandible than those with an isolated RS. It is speculated that in children with RS, with an intrinsic tissue deficiency as primary cause, there is impaired growth of the mandible [20]. This may also explain why this group of children needed respiratory support more often. In contrast, in the case of isolated RS, there might be catch-up growth from an initial deformation [21]. However, there is an ongoing discussion about the concept of accelerated growth of the mandible. Previous studies showed a “partial mandibular catch-up growth” and an increase (3.5 times its original size) in airway dimension in the first 2 years of life. Additionally, an increase in upper airway dimensions in longitudinal cephalograms of children with RS from childhood to adulthood was noticed [22, 23]. Remarkably, the depth of the oropharyngeal airway was an exception to this. In contrast, other studies did not observe this acceleration of mandibular growth [24–26]. Since the upper airway is a three-dimensional and complex dynamic altering space, one may question whether the method of analyzing two-dimensional radiographs (cephalogram) to determine alterations of airway space is the most appropriate one.

Ultimately, flexible fiberoptic laryngoscopy (FFL) of the upper airway should determine the extent of the upper airway obstruction, but validated scoring systems that objectify the obstruction of the airway are lacking. A recent attempt to score glossoptosis in RS patients using awake FFL was not successful, due to disappointing inter- and intra-agreement in the analysis of awake FFL videos of RS patients compared to non-RS patients [27]. Another complicating factor is that the degree of micrognathia does not seem to correlate well with the degree of airway compromise and the higher Cormack–Lehane grades diagnosed with laryngoscopy [28].

### Limitations

Only about half the RS population participated, resulting in a small sample size. Of some non-participants, there were no data available on the further course of the obstructive problems. Nonetheless, a flowchart was created based on the latest available data in the patient charts. In Table 1, it is shown that our sample appears to be representative of the total RS population in our clinic.

A PSG or appropriate PSG results were not available in all participants. If there was no PSG result available at cross section, the presence of OSA was determined by the child’s current need for respiratory support. Because of this, results from this study should be interpreted with caution.

Furthermore, our inclusion was based on historical data, which is another limitation of this study. Especially, in the group who was initially treated by prone positioning, one may argue whether these children are “true” Robin sequence patients. What was the indication to start prone positioning? What was the level of airway obstruction? However, just the mere fact that these children were given a prone positioning advice despite knowing the risks such as sudden infant death syndrome suggests that the clinical issues must have been substantial.

## Conclusion

This is the largest cross-sectional study on OSA in children with RS to date. Half of the RS population had been treated with prone positioning, while the other half needed respiratory support. Children with RS, who were treated by prone positioning as an infant, appear to have a very low risk on obstructive pathology at a later age indicating some natural improvement. Children who needed respiratory support continued or re-developed dependence on respiratory support at a later age. Considering the potential long-term effects of untreated OSA, children with RS in which the airway obstruction cannot be managed with prone positioning only require close follow-up beyond the infant period preferably using PSG.
